# A Tetravalent Sub-unit Dengue Vaccine Formulated with Ionizable Cationic Lipid Nanoparticle induces Significant Immune Responses in Rodents and Non-Human Primates

**DOI:** 10.1038/srep34215

**Published:** 2016-10-05

**Authors:** Gokul Swaminathan, Elizabeth A. Thoryk, Kara S. Cox, Jeffrey S. Smith, Jayanthi J. Wolf, Marian E. Gindy, Danilo R. Casimiro, Andrew J. Bett

**Affiliations:** 1 Infectious Diseases and Vaccines, Merck Research Laboratories, Merck & Co., Inc., Kenilworth, NJ, USA; 2 Pharmaceutical Sciences, Merck Research Laboratories, Merck & Co., Inc., Kenilworth, NJ, USA; 3 Safety Assessment & Regulatory Affairs, Merck Research Laboratories, Merck & Co., Inc., Kenilworth, NJ, USA

## Abstract

Dengue virus has emerged as an important arboviral infection worldwide. As a complex pathogen, with four distinct serotypes, the development of a successful Dengue virus vaccine has proven to be challenging. Here, we describe a novel Dengue vaccine candidate that contains truncated, recombinant, Dengue virus envelope protein from all four Dengue virus serotypes (DEN-80E) formulated with ionizable cationic lipid nanoparticles (LNPs). Immunization studies in mice, Guinea pigs, and in *Rhesus macaques*, revealed that LNPs induced high titers of Dengue virus neutralizing antibodies, with or without co-administration or encapsulation of a Toll-Like Receptor 9 agonist. Importantly, LNPs were also able to boost DEN-80E specific CD4+ and CD8+ T cell responses. Cytokine and chemokine profiling revealed that LNPs induced strong chemokine responses without significant induction of inflammatory cytokines. In addition to being highly efficacious, the vaccine formulation proved to be well-tolerated, demonstrating no elevation in any of the safety parameters evaluated. Notably, reduction in cationic lipid content of the nanoparticle dramatically reduced the LNP’s ability to boost DEN-80E specific immune responses, highlighting the crucial role for the charge of the LNP. Overall, our novel studies, across multiple species, reveal a promising tetravalent Dengue virus sub-unit vaccine candidate.

Dengue virus, the causative agent of Dengue fever (DF), Dengue Hemorrhagic Fever (DHF), and Dengue Shock Syndrome (DSS) belongs to the Flaviviridae family of RNA viruses. Other notable flaviviruses are West Nile Virus, Hepatitis C Virus and Zika virus, which are important pathogens of global concern^1^. Each year, it is estimated that about 390 million humans are infected with Dengue virus, making it one of the fastest expanding mosquito-borne infections world wide^2^. In spite of several years of research, no specific anti-viral inhibitors against Dengue virus have been approved for clinical use^3^,^4^. Numerous Dengue vaccine candidates have been evaluated in the preclinical and clinical settings, resulting in varying degrees of protective efficacy^5^. There are four related, yet distinct serotypes of Dengue virus (DENV-1, DENV-2, DENV-3 and DENV-4). Natural infection of humans with one serotype of Dengue virus is believed to confer lifelong protection against re-infection by the same serotype. However, pre-existing immunity to one serotype of Dengue virus is thought to exacerbate the outcome in humans infected with a different serotype, resulting in life threatening illness such as DHF or DSS. Therefore, the need for developing a tetravalent Dengue vaccine formulation, that will provide durable immunity against all four serotypes of the virus, is a crucial factor in the development of a successful vaccine^6^.

Currently, two leading Dengue vaccine candidates that have shown significant clinical success are live-attenuated, viral vectored, tetravalent vaccines^7^. Sub-unit vaccines are considered to be safer than live-attenuated/viral vectored vaccines^8^. But, sub-unit vaccines typically induce inferior B-cell and CD4+ T cell responses in comparison to live-attenuated vaccines and often do not yield CD8+ T-cell responses^9^,^10^. Development of a sub-unit Dengue vaccine has focused on the Dengue virus envelope (env) as the antigen, formulated with various vaccine adjuvants^11^,^12^. The main goal of this vaccine effort is to elicit Dengue virus neutralizing antibodies (nAb), since the env protein serves as the primary target of neutralizing antibodies in natural Dengue virus infections^11^,^13^,^14^. The live-attenuated Dengue vaccine strategies either utilize chimeric viral vectors that express Dengue virus envelope proteins^15^,^16^ or uses live-attenuated Dengue virus backbone of one serotype to express proteins of all four Dengue serotypes^17^,^18^. These vaccines generate both B-cell (nAb) and T-cell responses. There has been some debate over the immune correlates needed for protection against Dengue virus^19^. Research from murine models of Dengue virus infection, and analyses from naturally infected humans, have established a need for T-cell responses, particularly CD8+ T cell responses against Dengue virus^20^,^21^. However, long-standing evidence in the literature provides stronger evidence for the absolute necessity of Dengue virus neutralizing antibodies^22^,^23^. Therefore, a significant advancement in the Dengue vaccine field would be the development of a safe, sub-unit vaccine that can generate Dengue specific neutralizing antibodies, CD4+ T cells and CD8+ T cell responses. One major obstacle in the successful development of a sub-unit vaccine against Dengue virus is the poorly immunogenic nature of Dengue envelope proteins (as antigen). We have previously described the design and evaluation of recombinant, truncated, Dengue envelope antigens (DEN-80Es), and the immune responses elicited when delivered with various adjuvants^13^. Consistently the vaccine adjuvant that has yielded the best neutralizing antibody responses (as compared to other evaluated adjuvants), in murine and non-human primates, is the saponin based ISCOMATRIX™ adjuvant^14^.

Lipid Nanoparticles (LNPs) have dramatically influenced the field of vaccination: both as efficient delivery vehicles of innate immune agonists/adjuvants and as vaccine antigen delivery systems^24^. We have evaluated the vaccine adjuvant properties of specific cationic LNPs and recently reported that the Merck Lipid Nanoparticles have potent adjuvant properties when combined with Hepatitis B Virus Surface antigen (HBsAg) and Ovalbumin (OVA) in BALB/c and C57BL/6 mice vaccination studies. Interestingly, the adjuvant properties of LNPs did not require the need for co-administering known innate immune agonists^25^. Therefore, we investigated the impact of formulating ionizable cationic LNPs with Dengue envelope (DEN-80E) protein(s). Here, we report extensive preclinical evaluation of immunogenicity and safety, which demonstrate that LNP containing Dengue sub-unit vaccine formulations are well-tolerated, and elicit strong DEN-80E specific B-cell and T-cell responses in rodents and non-human primates.

## Results

### Lipid Nanoparticles enhance Dengue-2 envelope protein specific antibody responses in mice that possess virus neutralizing activity

We evaluated the ability of Merck LNPs to boost immune responses to a recombinant, truncated envelope protein from Dengue virus (DENV) serotype 2 (DEN2-80E) In previously reported preclinical studies evaluating DEN-80E antigens, formulations containing ISCOMATRIX^TM^ adjuvant were found to result in the strongest DENV neutralizing antibody titers in both mice and non-human primates, and therefore it was selected as the comparator in the current studies^13^,^14^ DENV’s envelope specific antibodies that possess neutralizing activity are currently the main focus of Dengue vaccine clinical trials^26^

ISCOMATRIX^TM^ adjuvant contains ISCOPREP^TM^ saponin, a purified fraction of Quillaia saponin, cholesterol and phospholipid^27^. Published studies suggest that the ISCOMATRIX^TM^ adjuvant results in the induction of both Th1 and Th2 type responses^27^. The mechanism of action of ISCOMATRIX^TM^ adjuvant is reported to involve a MyD88 dependent pathway for induction of CD8+ T cell responses^28^, as well, as inflammasome dependent and independent mechanisms for its *in-vivo* functionality^29^.

**Figure 1:**
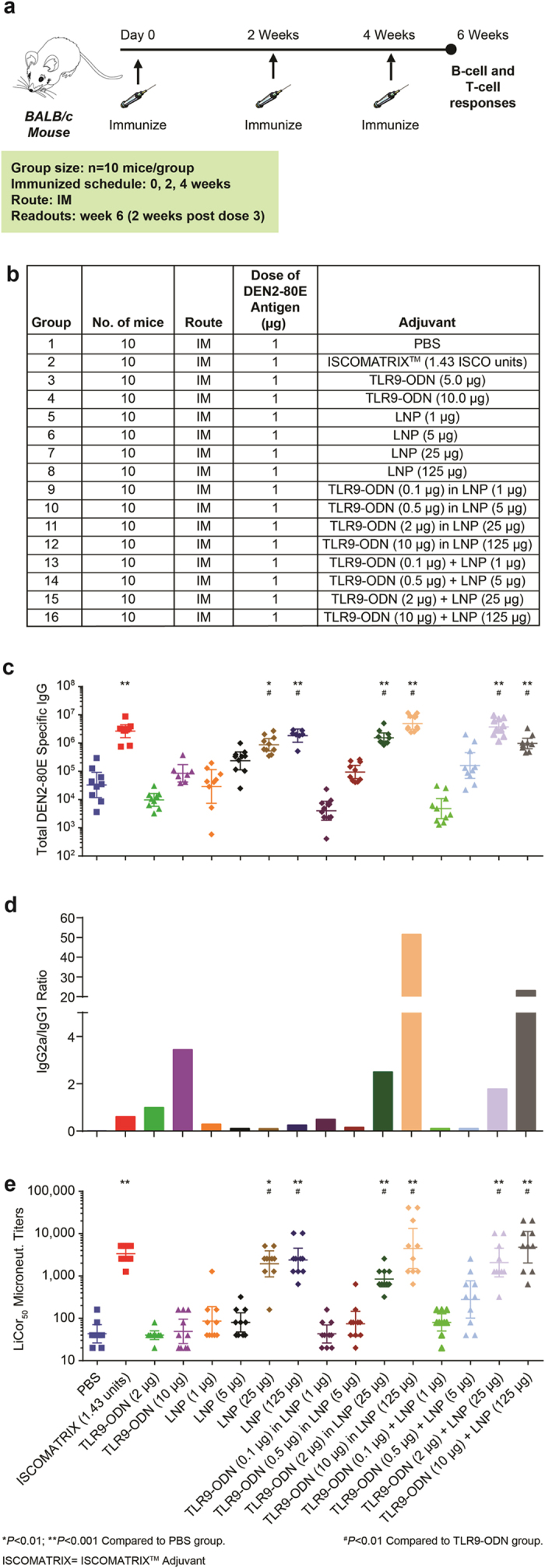
Lipid Nanoparticles significantly increases DEN2-80E specific humoral immune responses in mice. The immunization regime and dosing schedule for BALB/c mice vaccinated with 1 μg of DEN2-80E antigen, formulated with various adjuvants are depicted (**a,b**). DEN2-80E antigen was not encapsulated within LNP, but, merely co-mixed and administered intramuscularly as specified. Total IgG responses to DEN2-80E determined by ELISA in DEN2-80E vaccinated mice with LNP by itself, LNP co-administered with, or encapsulating TLR9-ODN (**c**). Ratio of DEN2-80E specific IgG2a to DEN2-80E specific IgG1 titers determined from pooled mice sera from each group (**d**). Dengue virus serotype 2 specific neutralizing activity in individual immunized mouse sera was calculated using the LiCor based MicroNeut assay (**e**). All assays were performed in sera collected at 2 weeks post-dose 3, in duplicates, from 10 mice/group. *p < 0.01 and **p < 0.001 denote statistical significance compared to PBS group and #p < 0.05 denotes statistical significance compared to TLR9-ODN (only) group.

For murine studies, mice (n = 10 /group) received, 3 doses (one prime and two boosts) of DEN2-80E (1 μg) formulated with PBS or the specified adjuvant, at 2-week intervals, (Fig. 1a). DEN2-80E antigen was not encapsulated within the LNP, but merely co-mixed prior to intramuscular injection. The LNPs were evaluated both alone and in combination with TLR-ODN. 2 weeks after the final dose, DEN2-80E-specific total serum IgG antibody titers were determined by end-point dilution ELISA. As expected, a statistically significant (p < 0.001) increase in DEN2-80E specific total IgG responses was observed in mice that received ISCOMATRIX^TM^ adjuvant compared to the PBS control group (Fig. 1b). In contrast, a significant increase in titers was not seen in mice that received TLR9-ODN (2 μg and 10 μg). When TLR9-ODN was co-administered with or encapsulated within LNP, a dose depended increase in total IgG titers was observed with the two highest dose groups of TLR9-ODN (2 μg and 10 μg) formulated with LNP (25 μg and 125 μg) demonstrating statistical significance (p < 0.01) over the PBS group. Notably, mice that received 25 μg or 125 μg of LNP alone, showed statistically significant (p < 0.01) increases in DEN2-80E specific IgG response, suggesting these responses could be driven by LNPs themselves. Further isotyping of IgG antibodies was carried-out by assaying DEN2-80E specific IgG1 (Th2 biased) and IgG2a (Th1 biased). The isotope profile (IgG2a:IgG1 ratio) of antigen specific IgGs has been established as a marker to assess Th1 vs Th2 type immune response bias. Inspite of comparable total IgG titers, co-administration or encapsulation of TLR9-ODN (2 μg and 10 μg) with LNP (25 μg and 125 μg) led to a more Th1 type antibody response. In contrast, the LNP only immunized mice showed a Th2 type antibody profile (Fig. 1c). In addition to evaluating the total quality and quantity of DEN2-80E specific IgG responses in immunized mice, we evaluated the functional antibody response using a LiCor based DENV neutralization assay (as described in materials & methods). In this assay, sera from immunized mice were pre-incubated at various dilutions with DENV serotype 2 (DEN2) before adding the mixture to vero cells and calculated the neutralization titers (NT50). As expected, the group that received ISCOMATRIX^TM^ adjuvant yielded statistically significant levels of DEN 2 virus neutralization titers (Fig. 1d). Notably, LNP by itself at 25 μg and 125 μg, or TLR-ODN (2 μg and 10 μg) co-administered or encapsulated with LNP (25 μg and 125 μg), also resulted in statistically significant DEN2 virus neutralization titers (nAb). Overall, these data strongly suggest that LNP adjuvant by itself was able to induce strong DEN2-80E specific antibody responses that were functionally effective at neutralizing Dengue Virus 2 infection.

### DEN2 env specific CD4+ and CD8+ T cell responses are enhanced by Lipid Nanoparticles

**Figure 2:**
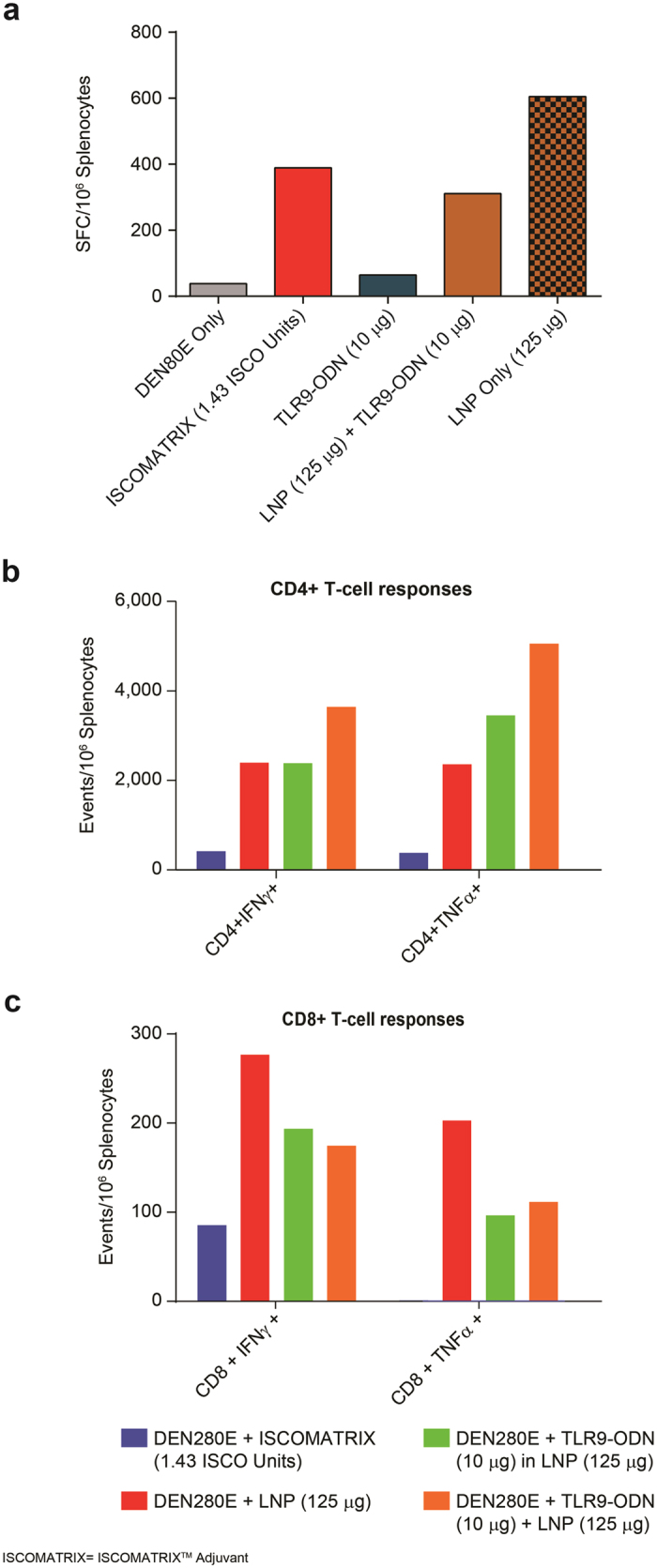
T cell responses to DEN2-80E in mice. 2 weeks after final vaccine dose, spleens from immunized mice were collected, processed, and total DEN2-80E specific T cell response was evaluated. IFN-γ producing spots per million (SFU) splenocytes were determined by IFN-γ ELISPOT assay. Bars represent average value from pooled spleens from 5 randomly selected mice out of 10 immunized mice/group, after background subtraction from non-stimulated cells (**a**). DEN2-80E specific CD4+ T cells that express IFN-γ, and TNF-α (**b**), and DEN2-80E specific CD8+ T cells that express IFN-γ, and TNF-α (**c**) were evaluated by intracellular FACS assay (FACS ICS). Bars represent T-cell events per million splenocytes from 5 randomly selected mice out of 10 immunized mice/group after background subtraction from unstimulated cells.

Cell mediated immune responses were first evaluated by employing an IFN-γ ELISPOT assay. 2 weeks after the final vaccine dose (Fig. 1a), spleens from 5 randomly selected mice from each vaccination group were processed, and pooled splenocytes were incubated with overlapping peptides that cover the amino acid sequence of the DEN2-80E antigen. As compared to the un-adjuvanted DEN2-80E + PBS group, the number of IFN-γ+ spot forming units (SFUs), where significantly higher in the DEN2-80E + ISCOMATRIX^TM^ adjuvant group. As seen with the nAb titers, vaccination with TLR9-ODN + DEN2-80E vaccination did not result in a considerable increase in IFN-γ+ SFUs. However, co-administration of DEN2-80E with LNP (in the presence or absence of TLR9-ODN) dramatically enhanced the IFN-γ+ SFU formation, which demonstrated the LNP’s ability to enhance cell mediated immune responses to DEN2-80E antigen (Fig. 2a). Since ELISPOT assays do not distinguish between antigen specific CD4+ T cell or CD8+ T cell responses, we evaluated cell-mediated immune responses by flow cytometry intracellular staining (ICS) in pooled spleens from each group of vaccinated animals. The majority of the groups had undetectable levels of DEN2-80E specific T cell responses: mice vaccinated with DEN2-80E + PBS, DEN2-80E + TLR9-ODN, and the mice vaccinated with DEN2-80E + lower doses of LNP, LNP + TLR9-ODN and TLR9-ODN encapsulated in LNP. Vaccination with DEN2-80E + ISCOMATRIX^TM^ adjuvant resulted in minimal induction of antigen specific CD4+ and CD8+ T cells. However, vaccination with DEN-80E formulated with TLR9-ODN within LNP, LNP + TLR9-ODN, and LNP by itself (at the highest doses), yielded a substantial increase in DEN2-80E specific CD4+ T cells that express IFN-γ and TNF-α (Fig. 2b) and CD8+ T cell responses that express IFN-γ and TNF-α (Fig. 2c). The most significant frequency of DEN2-80E specific CD4 and CD8+ T cells were observed in the group that received the highest dose of LNP + TLR9-ODN + DEN2-80E. While LNP + DEN2-80E vaccination by itself was able to induce T cell responses, co-administration of TLR9-ODN significantly enhanced the frequency of both CD4+ and CD8+ T cell responses specific to DEN2-80E.

### Evaluation of cytokine and chemokine profiles in vaccinated mice

**Figure 3:**
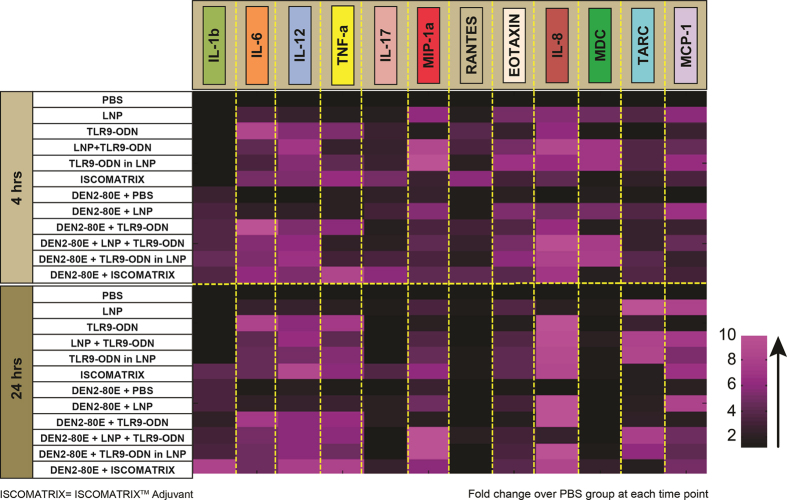
Cytokine & chemokine expression in immunized mice. Sera from mice immunized with PBS or LNP or specified adjuvants in the presence or absence of DEN2-80E vaccine antigen, were collected at 4 hrs and 24 hrs post dosing. Pooled mice sera from each group (n = 3), were assayed in triplicate. The average value of each cytokine and chemokine was calculated. The fold increases of cytokines & chemokines in treatment groups were calculated based on the PBS treated group at 4 hrs or 24 hrs time point (represented by the color code).

Having established the potency of DEN2-80E specific immune responses when combined with various adjuvants, we evaluated cytokine and chemokine profiles in vaccinated mice. Using a multiplex assay platform, we evaluated 16 cytokines and chemokines in pooled immunized mouse sera from each group, at two time points, 4 hrs and 24 hrs post intramuscular administration of adjuvant by themselves and in combination with DEN2-80E antigen. Shown in Fig. 3 is a heat map of fold change for each cytokine and chemokine as compared to PBS treated mice at each time point assayed. Mice injected with TLR9-ODN, TLR9-ODN + LNP, TLR9-ODN in LNP, and ISCOMATRIX^TM^ adjuvant by itself or in combination with DEN2-80E antigen, resulted in elevation of pro-inflammatory cytokines including IL-6 and TNF-α, and the Th1-type cytokine, IL-12. The inflammasome specific cytokine L-1β was increased in mice injected with DEN2-80E alone or in combination with other adjuvants, at both 4 hrs and 24 hrs post injection. This supports the notion that the Dengue envelope protein might be contributing to the inflammasome activation, previously reported upon *in-vitro* infection and in Dengue virus infected patients^30^,^31^. LNP alone, or in combination with DEN2-80E, did not induce significant levels of IL-1β and TNF-α, but a minimal increase in IL-6 was seen at 4 hrs and 24 hrs post injection. Other chemokines such as (i) Eotaxin that attracts eosinophils and neutrophils, and (ii) Interleukin-8 (IL-8), a chemokine that significantly attracts neutrophils and other innate immune cells, were significantly upregulated in sera of all mice injected with all adjuvants +/−antigen. However, macrophage specific chemokines such Macrophage Inflammatory Protein-1 (MIP-1a), Macrophage Derived Chemokine (MDC), and monocyte specific, Monocyte Chemotactic Protein-1 (MCP-1) were significantly increased in all LNP containing immunization groups.

### Reduction in ionizable cationic lipid content impacts LNP mediated increase in D2-80E specific responses in immunized mice

**Figure 4:**
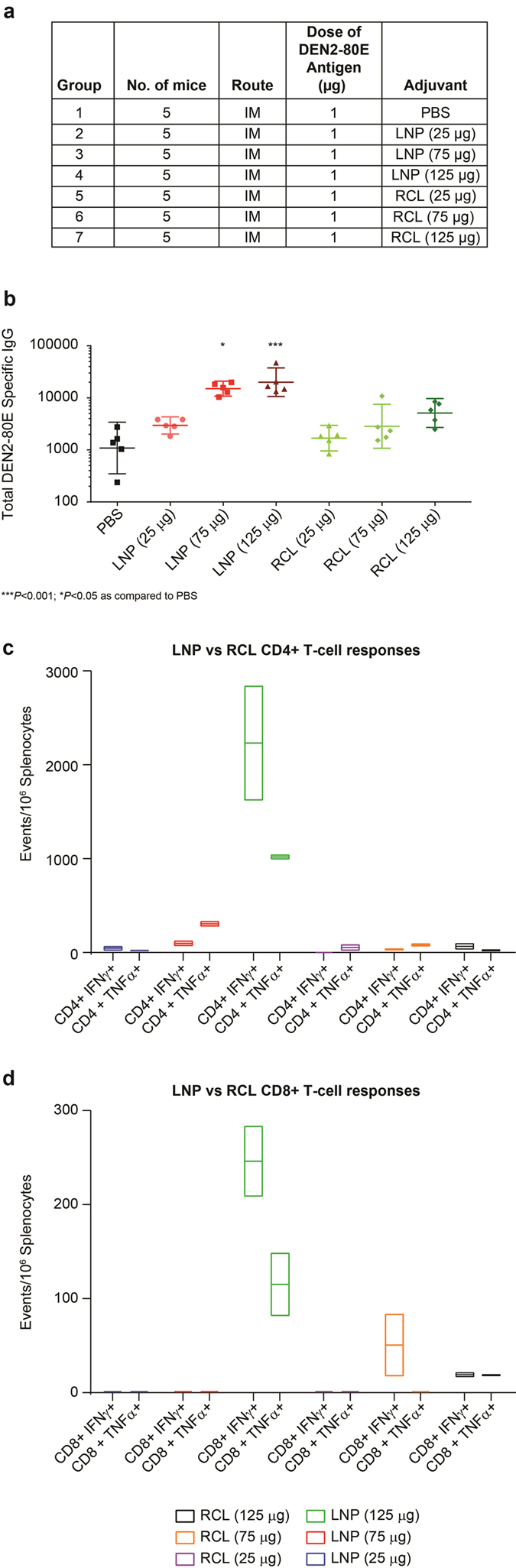
Reduction in cationic lipid content impacts LNP’s ability to boost DEN2-80E specific immune responses. The immunization regime and dosing schedule for BALB/c mice vaccinated with 1 μg of DEN2-80E antigen, formulated with PBS or LNPs (25 ug/75 ug/125 ug) or RCL (25 ug/75 ug/125 ug) are depicted (**a**). 2 weeks post dose 3, sera was assayed for DEN2-80E specific total IgG responses on an ELISA platform (**b**). 2 weeks post dose 3, spleens from all 5 mice/group were isolated, processed, and pooled to assess T cell responses. DEN2-80E specific CD4+ T cells that express IFN-γ, and TNF-α (**c**) and DEN2-80E specific CD8+ T cells that express IFN-γ, and TNF-α (**d**) were evaluated by intracellular FACS assay. Bars represent T-cell events per million splenocytes from 5 mice/group assayed in duplicate after non-stimulated subtraction. ***p < 0.001; *p < 0.05 as compared to PBS.

As described in our previous publications^32^,^33^, the lipid nanoparticle is composed of four main components: Merck proprietary ionizable cationic lipids, 1,2-distearoyl-glycero-3-phosphocholine (DSPC) phospholipid, cholesterol, and a PEGylated lipid, PEG (2000)-DMG. To specifically evaluate if reduction in the charge of the lipid nanoparticle would impact its ability to boost antigen specific immune responses, we manufactured LNPs, termed Reduced Cationic Lipid LNPs, (RCLs). RCLs have the same components as the original LNPs, but the content of ionizable cationic lipid is significantly reduced. We immunized BALB/c mice (n = 5) (similar vaccination regime as described in Fig. 1a) using DEN2-80E antigen formulated with three concentrations of LNP or RCL (25 μg, 75 μg and 125 μg) as adjuvants. As shown in Fig. 4a, a dose dependent increase in DEN2-80E specific IgG responses was observed in the LNP + DEN2-80E vaccinated groups, as compared to the DEN2-80E + PBS vaccinated group. While the 25 μg dose of LNP was insufficient, the 75 μg and 125 μg doses of LNP were sufficient to induce statistically significant increases in DEN2-80E specific total IgG responses. However, vaccination with DEN2-80E and RCL (25 μg, 75 μg and 125 μg) did not yield a statistically significant increase in DEN2-80E specific IgG titers (Fig. 4a). We next evaluated T cell responses by ICS in pooled spleens from all 5 mice per group as described in section 1.2. Evaluation of DEN2-80E specific CD4+ T cell response (Fig. 4b) and CD8+ T cell responses (Fig. 4c) revealed that LNP + DEN2-80E resulted in a dose dependent increase in detectable DEN2-80E specific CD4+ T cells and CD8+ T cells that expressed IFN-γ and TNF-α. In comparison, groups that received DEN2-80E + RCL had a lower frequency of DEN2-80E specific CD4+ T cell and CD8+ T cell responses. Overall, these data suggest that a reduction in the cationicity of the LNPs substantially reduced the LNP’s ability to boost B-cell and T-cell responses to DEN2-80E in vaccinated mice.

### LNP boosts neutralizing antibody responses against all four Dengue virus serotypes in Guinea pigs

**Figure 5:**
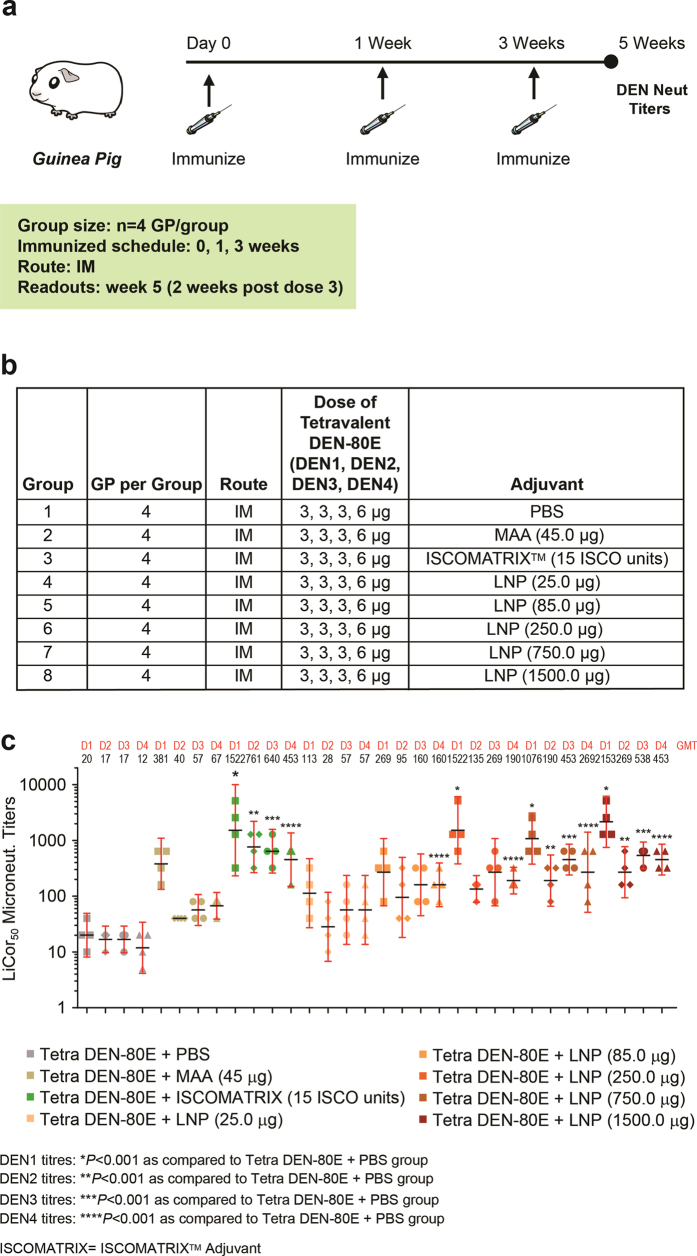
Dengue virus neutralization titers in Guinea Pigs Immunized with Tetravalent DEN-80E and LNP. The immunization regime, dosing schedule, and vaccine formulation details for Guinea pigs vaccinated with tetravalent Dengue vaccine (Tetra DEN-80E) is depicted (**a,b**). Dengue virus serotype 1 (D1), serotype 2 (D2), serotype 3 (D3), serotype 4 (D4) specific neutralizing activity from individual immunized Guinea pig sera was calculated using the LiCor based assay and depicted as LiCor microtneut NT50 titers (**c**). Each dot/symbol represents an individual animal. The geometric mean titers (GMT) of serotype specific neutralization are depicted on top of the graph above each group. DEN1 titers: *p < 0.001 as compared to DEN80E + PBS group; DEN2 titers: **p < 0.001 as compared to DEN80E + PBS group; DEN3 titers: ***p < 0.001 as compared to DEN80E + PBS group; DEN4 titers: ****p < 0.001 as compared to DEN80E + PBS group.

As previously mentioned, a successful Dengue vaccine should elicit neutralizing antibodies (nAbs) against all four serotypes of the virus. Therefore, building on our murine model using monovalent DEN2-80E + LNP, we evaluated if LNPs would be able to boost nAbs against all four DENVs in Guinea Pigs immunized with a tetravalent DENV-80E (DEN1-4-80E) vaccine. We utilized a pre-optimized dose of 3 μg, 3 μg, 3 μg, and 6 μg of DEN1-80E, DEN2-80E, DEN3-80E, and DEN4-80E respectively, formulated with varying doses of LNP (ranging from 25 μg to 1500 μg). Previous studies have determined that the DEN4-80E component is the least immunogenic and is therefore dosed at twice the concentration of the other three DEN-80E proteins in order to yield a more balanced nAb responses to all four serotypes. As controls, we utilized 15 units of ISCOMATRIX^TM^ adjuvant and 45 μg of Merck aluminum adjuvant (MAA). As depicted in Fig. 5a, Guinea pigs (n = 4) were vaccinated three times, two weeks apart, by the intramuscular route. 2 weeks after the last dose, sera from guinea pigs were collected and Dengue virus neutralization titles were determined. As shown in Fig. 5b, formulation with MAA did not significantly increase the nAb titers against any of the four DENV serotypes. Expectedly, ISCOMATRIX^TM^ adjuvant led to statistically significant increases in nAb titers against all four DENV serotypes. Formulation of LNPs with DEN1-4-80E resulted in a dose dependent increase in nAbs titers against all four serotypes, wherein the 750 μg and 1500 μg doses were statistically significant. This confirmed the potential for LNP’s to boost nAbs against all four serotypes of DENV when vaccinated with the tetravalent DEN1-4-80E antigens.

### Evaluation of LNP containing tetravalent Dengue vaccine in non-human primates

**Figure 6:**
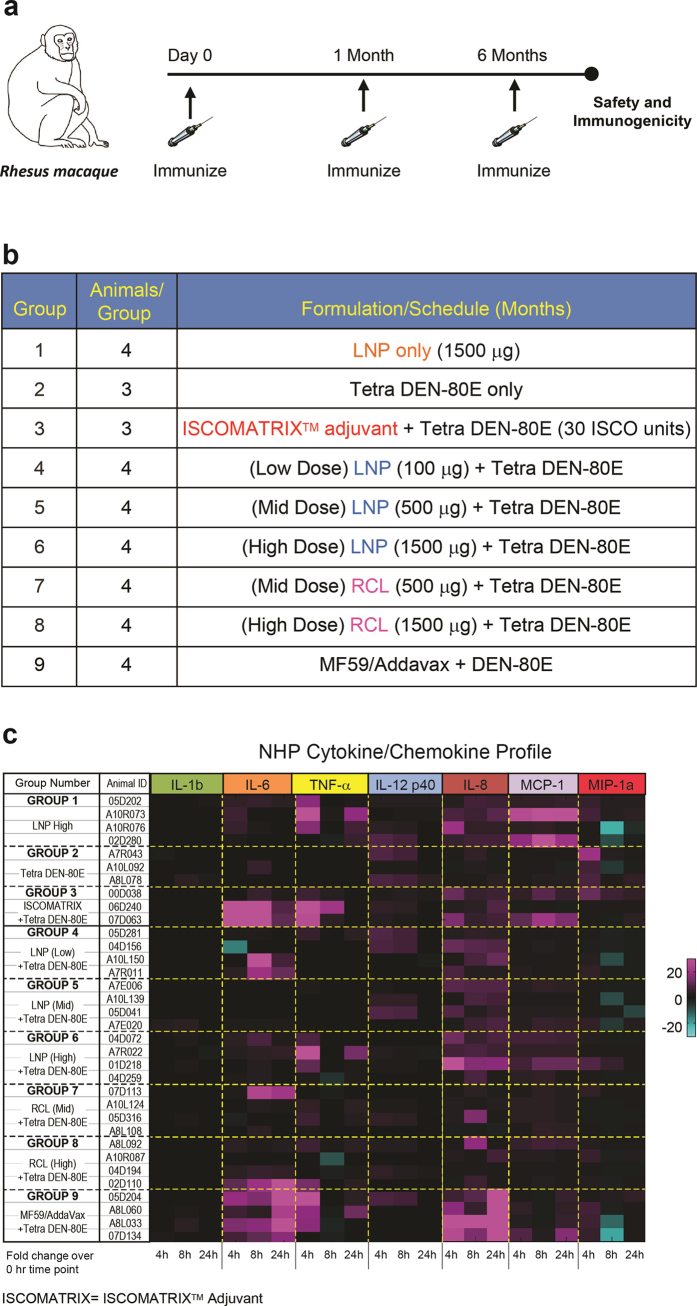
LNP containing Tetravalent Dengue sub-unit vaccine in Non-Human Primates. The immunization regime and dosing schedule for Rhesus macaques vaccinated using tetravalent Dengue vaccine (Tetra DEN-80E) is depicted (**a**). Group numbers, number of animals per group and the test formulation administered to each group is specified in Fig. 6b. Tetra DEN-80E vaccine contains 10 μg, 10 μg, 10 μg, 20 μg, corresponding to DEN1/DEN2/DEN3/DEN4 serotypes respectively. Sera from immunized Rhesus macaques with specified test agents were collected at 0 hrs, 4 hrs, 8 hrs, and 24 hrs post dosing. Individual sera from each group were assayed in duplicates using the Luminex platform. The average value of each cytokine/chemokine from immunized animals at 0 hr (prebleed) time point was used to calculate the fold change. The fold change at 4 hrs, 8 hrs, and 24 hrs of individual animals are based on the heatmap color code depicted in Fig. 6c.

Having confirmed that LNPs generated strong neutralizing antibodies against Dengue envelope antigen(s) in mice and Guinea pigs, and enhanced DENV env specific T-cell responses in mice, we investigated the safety and efficacy of this vaccine formulation in non-human primates. Specifically, we utilized a well-established *Rhesus macaque* model^14^. For this study animals received 10 μg, 10 μg, 10 μg, and 20 μg of the DEN1-80E, DEN2-80E, DEN3-80E and DEN4-80E antigens respectively formulated alone or with adjuvant. As outlined in Fig. 6a, the animals were vaccinated at 0, 1 and 6 months. The vaccination formulation administered to each group of animals is listed in Fig. 6b- each group contained 3–4, weight and sex matched animals. All vaccine formulations were injected intramuscularly, into the right deltoid. Using a Draize scoring scale from 0–4, (criteria are described in Supplementary Figure 1), animals were evaluated for the appearance of erythema and edema at the injection site for up to 7 days, post dose 1 (Fig. S1a), dose 2 (Fig. S1b), and dose 3 (Fig. S1c). No vaccination-induced local site reactogenicity was observed in any of the vaccine groups, including animals that received the highest dose of LNP and RCL, suggesting that the Dengue vaccine formulation containing LNP was well tolerated (Supplementary Figure 1).

Using a 23-plex Luminex panel, we evaluated cytokine and chemokine profiles in sera from immunized animals, prior to dosing (0 hr) and at 4, 8, 24 and 48 hrs post dose 1. The majority of the cytokines and chemokines showed no significant changes at 48 hrs post injection (data not shown). Shown in Fig. 6c are fold changes of key cytokines and chemokines in each animal at 4, 8, and 24 hrs, calculated over the 0 hr time point of each animal. We chose to focus on the cytokines and chemokines that showed more consistent trends between animals within a group and the ones of biological relevance. The inflammasome specific cytokine IL-1β, was not significantly upregulated in any treatment group including in the LNP (high dose) treated animals, unlike what was observed in mice. The pro-inflammatory cytokine, IL-6 was mildly upregulated in most treatment groups including in the LNP (High) group, with or without DEN1-4-80E. However, the levels were far less significant as compared to the sustained upregulation observed in animals in the ISCOMATRIX^TM^ adjuvant + DEN1-4-80E group (Group 3) and MF59/AddaVax^TM^+DEN1-4-80E group (Group 9). Sera from animals in the LNP high dose (group 1), LNP(High) + DEN1-4-80E (Group 6), RCL + DEN1-4-80E (Mid and High) (Groups 7 and 8), MF59/AddaVax + DEN1-4-80E (Group 9) displayed an increase of the pro-inflammatory cytokine, TNF-α at 4 hrs. The Th1 type cytokine IL12 was also mildly upregulated in LNP containing vaccine formulations. The chemokine IL-8 was highly upregulated in all vaccination groups except the DEN1-4-80E only (Group 2). The monocyte chemotactic protein (MCP-1) was significantly upregulated in LNP (High) (Group 1) and LNP(High) + DEN1-4-80E (Group 6) at 4, 8, and 24 hrs, whereas DEN1-4-80E mixed with ISCOMATRIX^TM^ adjuvant and MF59/AddaVax^TM^ also led to increase in MCP-1.

In addition to cytokines and chemokines, a variety of safety biomarkers were evaluated in sera samples collected from immunized animals at day 1, 3 and 7-post dose 1 (PD1). Evaluation of Alanine Transaminase (ALT) (Fig. S2a), Aspartate Transaminase (AST) (Fig. S2b), Total bilirubin (Tbil) (Fig. S2c), Direct bilirubin (Dbil) (Fig. S2d), and C-Reactive Protein (CRP) (Fig. S2e), in sera samples at day 1, post-dose 1 revealed no significant elevation in immunized animals. These biomarkers were unaltered at day 3 and day 7 (PD1) samples as well (data not shown). Assessment of blood lipid content (lipemia), hemolytic activity and icteric activity showed no elevation post vaccination (data not shown). Furthermore, we evaluated C3a and CBb levels as a measure of complement activation in immunized animals (Supplementary Figure 3). Measurement of C3a (S3a) and CBb (S3b) in plasma samples from immunized animals at 0, 4, 24 and 72 hrs, post dose 3, demonstrated that LNP or RCL injection does not lead to a significant level of complement activation. Overall, through the exhaustive list of biomarkers evaluated, we conclude that the LNP containing Dengue vaccine formulations did not increase the level of biomarkers associated with liver injury, systemic inflammation, or complement activation.

### LNP containing tetravalent Dengue vaccine formulation enhances Dengue Virus Neutralizing Antibodies in Rhesus macaques

**Figure 7:**
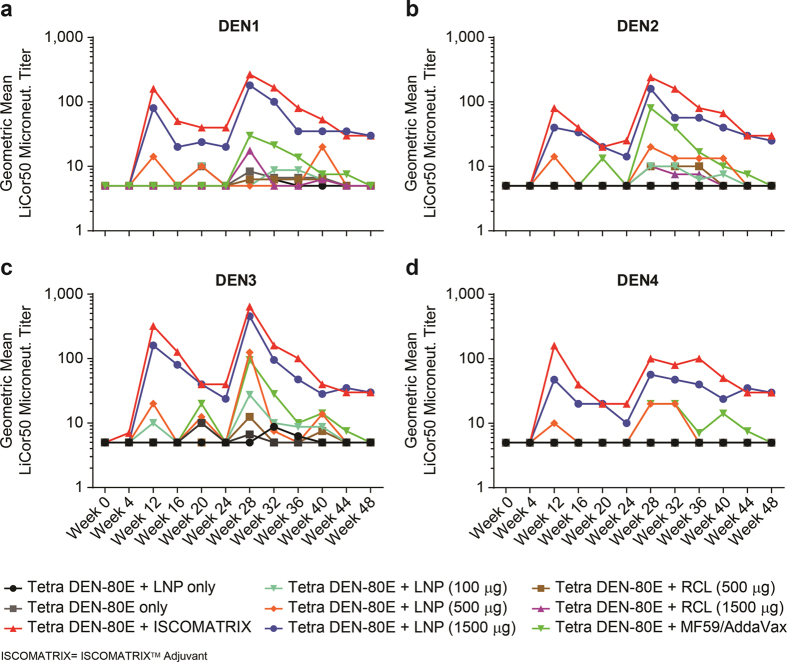
Longitudinal Evaluation of Dengue Virus Neutralizing Antibodies in immunized Rhesus macaques. Longitudinal geometric mean virus neutralization titers in sera derived from immunized animals with various Tetra DEN-80E vaccine formulations. Virus neutralizing antibody titers against Dengue virus serotype 1 (**a**), serotype 2 (**b**), serotype 3 (**c**), and serotype (**d**), were measured over a period of 48 weeks using a LiCor-based neutralization assay. Each dot/symbol represents an individual animal.

Having established safety profiles, the magnitude and durability of neutralizing antibodies (nAb) against each of the four Dengue serotypes was evaluated in serum samples from vaccinated animals. The LiCor-based micro-neutralization assay was used to determine nAb titres against all four serotypes Fig. 7a–d), every 4 weeks, longitudinally, out to 48 weeks from the initial vaccination date. As previously observed formulation of DEN1-4-80E with the ISCOMATRIX^TM^ adjuvant (group 3) resulted in the highest geometric mean nAb titres against all four serotypes^14^. As observed in Guinea pigs, the nAb titres in NHPs that received LNP high + DEN1-4-80E (group 6) were comparable to those seen in the ISCOMATRIX^TM^ adjuvant group (red line), and significantly higher than titres observed in the DEN1-4-80E + PBS (Group 2) or DEN1-4-80E + MF59/AddaVax^TM^ (group 9) vaccinated animals. The responses were directly proportional to the dose of LNP used i.e. nAb titres were highest in animals that received the high dose (red line), followed by mid dose (orange line), and then low dose (cyan line) of LNP + DEN1-4-80E. Interestingly, as seen in mice, a reduction in the cationicity of the LNP (RCL), inhibited the LNP’s ability to enhance nAb titres. The magnitude of nAbs titers to DEN1-4-80E vaccination waned over time, as seen at week 24 prior to the third dose. However, measurement of nAb titres 4 weeks after the third vaccine dose (week 28), showed a significant increase in nAb titres against all four serotypes both in group 3 and group 6. Interestingly, DEN1-4-80E + MF59/AddaVax^TM^ (group 9/green line) also showed a modest increase in nAbs against all four Dengue serotypes, inspite of undetectable responses before the third dose. The nAb titres obtained from group 3 and group 6, declined slightly following the third dose, but were still detected out to week 48 (~6 months) after the last dose. Overall, these results demonstrate that LNP (High) + DEN1-4-80E vaccination led to significant nAb titres against all four Dengue virus serotypes, comparable to DEN1-4-80E+ ISCOMATRIX^TM^ adjuvant (group 3), and significantly higher than all other vaccine formulations.

### DEN2 env specific CD4+ and CD8+ T cell responses are enhanced by Lipid Nanoparticles

**Figure 8:**
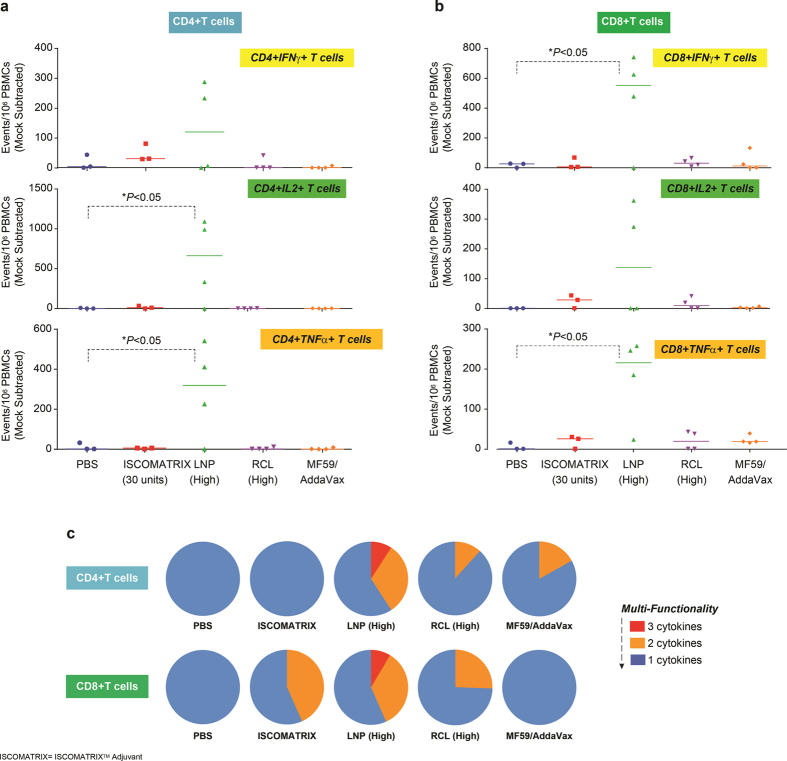
LNP increases T-cell responses in Tetra DEN-80E vaccinated Rhesus macaques. At week 28 (4 weeks after final vaccine dose), Peripheral Blood Mononuclear Cells (PBMCs) were collected from immunized animals and incubated with DEN1-80E, DEN2-80E, DEN3-80E, DEN4-80E specific peptide pools, and subjected to FACS ICS analysis. DEN4-80E specific CD4+ T cells that express IFN-γ, IL-2, and TNF-α (**a**) and DEN4-80E specific CD8+ T cells that express IFN-γ, IL-2, and TNF-α (**b**) were calculated as T-cell events per million PBMCs. Each dot/symbol represents an individual animal. The multi-functionality of the T-cell responses was calculated using the SPICE software and represented here as pie charts. Out of the three cytokines assessed (IFN-γ, IL-2, and TNF-α) in antigen specific T-cells, expression of one cytokine by T-cells from a vaccinated group is assigned blue, two cytokines is assigned orange and three cytokines is assigned red (**c**). *p < 0.05 as compared to DEN80E + PBS group.

In addition to evaluating nAb in the vaccinated Rhesus macaques (Fig. 6b), we evaluated T cell responses. 4 weeks post-dose 3 (Week 28), Peripheral Blood Mononuclear Cells (PBMCs) were collected from all vaccinated animals. One million PBMCs from each animal were individually incubated with peptides pools representing DEN-80E protein from each of the four serotypes. Using a FACS ICS assay, CD3 + CD4+ T cells and CD3 + CD8+ T cells that expressed cytokines such as IFN-γ, IL-2, and TNF-α specific to DEN1-80E, DEN2-80E, DEN3-80E, and DEN4-80E were measured. Surprisingly, no significant increase in CD4+ or CD8+ T cell responses specific to DEN1-80E, DEN2-80E, DEN3-80E antigens were observed in any immunized animals. However, both CD4+ and CD8+ T cells were detected against DEN4-80E antigen in some immunized animals. Shown in Fig. 8 are DEN4-80E specific responses in animals immunized with 1) PBS + DEN1-4-80E (Group 2- no adjuvant), designated as PBS; 2) ISCOMATRIX^TM^ adjuvant + DEN1-4-80E (Group 3), designated IMX; 3) LNP (High) + DEN1-4-80E (Group 6), designated LNP (high); 4) RCL (High) + DEN1-4-80E (Group 7), designated RCL (high); 5) MF59/AddaVax^TM^ + DEN1-4-80E (Group 9), designated MF59/AddaVax^TM^, where each dot represents an individual animal. Vaccination of LNP (High) + DEN1-4-80E (green triangles) resulted in a significant increase in DEN4-80E specific CD4+ T cell responses (Fig. 8a) and CD8+ T cell responses (Fig. 8b) that were positive for IFN-γ, IL-2, and TNF-α. All the other vaccinated animals, including, groups containing ISCOMATRIX^TM^ adjuvant or MF59/AddaVax^TM^ with DEN1-4-80E antigen yielded minimal levels of detectable CD4+ T cell or CD8+ T cells responses. The multifunctional quality of the T cell responses from this assay was also evaluated i.e. DEN4 specific individual T cells that express one, two or all three cytokines. As depicted as piecharts in Fig. 8c, only LNP (high) + DEN1-4-80E vaccinated animals showed DEN4-80E specific multi-functional CD4+ and CD8+ T cell responses that expressed all three cytokines-IFN-γ, IL-2, and TNF-α. Interestingly, as seen in mice, RCL (high) + DEN1-4-80E did not result in a significant increase in DEN4 specific immune responses, further confirming that the ionizable cationic lipid content of LNPs is a crucial factor needed to boost antigen specific T cell responses (LNP vs RCL). We have previously reported that ISCOMATRIX^TM^ adjuvant + DEN1-4-80E vaccination in Non-Human Primates induces appreciable amounts of antigen specific T cell responses [28]. Therefore, to further confirm the T cell responses assayed via FACS ICS (Fig. 7), we analyzed PBMCs collected from animal at week 28 and week 40, from group 2, group 3, and group 6 in an IFN-γ ELISPOT assay after incubation with DEN1/2/3/4-80E specific peptide pools. The ELISPOT assay is considered more sensitive than the FACS ICS assay, therefore, this served as an ideal confirmatory assay. The number of IFNγ+ spot forming units (SFUs) per million cells was mock subtracted (DMSO treatment) and graphed in Supplementary Figure 4a (week 28) and Supplementary Figure 4b (week 40). At both time points, IFNγ+ SFU specific for DEN1, DEN2, DEN3, and DEN4 were undetectable in the PBS group, minimally detectable in ISCOMATRIX^TM^ adjuvant + DEN1-4-80E group, but readily detected in LNP (high) + DEN1-4-80E immunized animals. Confirming the FACS ICS data, the DEN4-80E specific responses were much higher in the LNP (high) + DEN1-4-80E vaccinated group as compared to DEN1/2/3-80E antigens. Overall, these data suggested that LNPs have the potential to enhance cell-mediated immune responses to a sub-unit recombinant antigen in non-human primates.

## Discussion

A successful Dengue vaccine that protects against all four serotypes of Dengue viruse is urgently needed. Preclinical and clinical evaluation of sub-unit and live attenuated Dengue vaccines has met with varying degrees of safety and immunogenic protection^34^,^35^. Most recently, Sanofi Pasteur’s recombinant, live, attenuated Chimeric Yellow Fever-Tetravalent Dengue Vaccine (CYD-TDV), that expresses preM and env proteins of Dengue virus, became the first Dengue vaccine to be approved for human use for the prevention of Dengue virus infection by two countries; Mexico^36^ and Philippines^37^. Clinical trials in Asia and in Latin America reported that Dengue serotype 1, 3 and 4 specific seroconversion was successfully induced in individuals who received this vaccine, but insufficient seroconversion was observed for Dengue serotype 2^38^,^39^,^40^,^41^.

Live-attenuated, viral vectored Dengue vaccines can result in an imbalance of equipotent serotype specific immune responses and thereby impacts vaccine-induced protection against all four serotypes of Dengue virus^42^. This issue can be circumvented with a subunit vaccine where one can strategically manipulate the Dengue antigen content/concentration included in the formulation. For instance, extensive research from our group has demonstrated that doubling the amount of recombinant envelope protein for Dengue virus serotype 4 in the sub-unit vaccine formulation compared to the env protein concentration of serotypes 1, 2 and 3, is required for generating a more balanced neutralizing antibody (nAb) response against all four serotypes in murine and non-human primate vaccination studies^14^. In addition, sub-unit Dengue vaccines offer substantial advantage over live attenuated vaccines since they are safer (to vaccinate immune suppressed and pediatric population), and provide long-term vaccine stability (low risk of genetic mutations).

Generation of nAbs against all four Dengue viruses has been a key objective of current Dengue vaccine candidates^19^. However, emerging evidence suggests that T cell responses are also crucial for protection against Dengue virus infection^43^. Based on our recent discovery that specific ionizable cationic lipid nanoparticles (LNPs) can boost B-cell and T-cell responses to sub-unit antigens, we evaluated a novel tetravalent subunit Dengue virus vaccine formulation containing ionizable cationic LNPs. Here we report that ionizable cationic LNPs significantly enhanced the generation of nAbs to dengue envelope proteins in mice, Guinea pigs, and *Rhesus macaques*, to levels comparable to the well-characterized ISCOMATRIX™ adjuvant.

The lack of reliable animal models for Dengue virus infection and disease outcomes has been a significant limiting factor in the field^44^. Evaluation of safety and immunogenicity of Dengue vaccine candidates have largely relied on the use of Rhesus macaques. However, the Rhesus macaque model does not recapitulate the disease outcome of Dengue virus infection as observed in humans. We have extensively reported that nAbs titres induced upon DEN1-4 80E + ISCOMATRIX™ adjuvant vaccination is sufficient to reduce viremia against Dengue virus challenge^14^,^45^. LNP (High) + DEN1-4-80E vaccination induced antibody titers that were comparable to DEN1-4-80E + ISCOMATRIX™ adjuvant vaccination in quality (neutralizing all four serotypes of Dengue viruses) and durability. Therefore, we chose not to challenge the LNP + DEN1-4-80E immunized animals, as we fully expect that the animals would have no detectable viremia. In addition, we would not be able to investigate the impact of LNP + DEN1-4-80E vaccination on Dengue virus induced disease severity.

The evaluation of cell mediated immune responses in mice and Rhesus macaques revealed that the LNPs were far superior to ISCOMATRIX™ adjuvant in generating poly-functional CD4+ T cell responses and CD8+ T cell responses in immunized animals. In mice, we observed that the frequency of DEN2-80E specific CD8+ T cell responses, but not CD4+ T cell responses, was slightly reduced in groups that received formulations containing LNP and TLR9-ODN (Fig. 2c). This unique observation warrants extensive investigation of the specific subsets of innate immune cells that uptake the antigen upon co-administration with LNP *versus* LNP + TLR9-ODN. We hypothesize that cell types that are recruited to the injection site, uptake antigen and migrate to the local draining lymph nodes may be different between LNP and TLR9-ODN containing groups. In particular, the cross-presenting innate immune cells such as the CD8+ dendritic cells. In Rhesus macaques, inspite of minimal DEN-80E specific CD4+ T cell responses induced upon DEN-80E + ISCOMATRIX™ adjuvant and DEN-80E + LNP (high) vaccination, we observed significant Dengue virus specific nAb titers. However, it has been established in the literature that CD4+ T cells are not required for generation of Dengue vaccine specific nAb or CD8+ T cell responses^46^. In LNP (high) + DEN1-4-80E vaccinated non-human primates, out of the four antigens (DEN1-4-80E), we only observed CD8+ T cells responses to DEN4-80E. This may be because the MHC-I specificity for Dengue antigens has not been characterized in *Rhesus macaques*. Furthermore, the Dengue virus non-structural proteins NS1, NS3 and NS5 have been characterized to be the main epitopes of CD8+ T cell responses in humans^47^ and Cynomolgus macaques^48^ vaccinated with live-attenuated Dengue virus vaccines. Therefore, inclusion of NS1, NS3 and NS5 Dengue virus non-structural proteins, as additional antigens in the LNP + DEN1-4-80E vaccine formulation might be beneficial.

The ability of our ionizable cationic LNPs to boost DEN-80E specific CD8+ T cell responses in mice and, more importantly, in non-human primates is of utmost significance. Generation of CD8+ T cell responses to recombinant protein antigens in non-human primates is exceptionally rare. To our knowledge, the only known published report demonstrating this effect required the direct conjugation of a Toll-Like Receptor 7/8 (TLR7/8) agonist to the protein antigen^49^. Here, we report that the LNP has the ability to induce multi-functional, CD8+ T cell responses (Fig. 8b) without the need for direct conjugation to the protein. This offers significant manufacturing advantages to the vaccine formulation. More importantly, there was no requirement for inclusion of any known innate immune agonists in the vaccine formulation to yield significant B-cell or T-cell responses against the antigen.

We postulate that the LNP has strong immune-modulatory properties, possibly enabling the activation innate immune pathways. Although not experimentally confirmed at this time, we believe that our LNP is able to induce such strong CD8+ T cell responses through its ability to either specifically deliver the co-administered antigen to cross-presenting DCs or through activating these cells to enhance antigen presentation. Furthermore, we are committed to uncovering the mechanism of action behind the LNP’s ability to boost such robust B-cell, CD4+ T cell and more specifically, CD8+ T cell responses to sub-unit antigens. It has been reported that lipid nanoparticles can activate specific Toll-Like Receptor pathways^50^,^51^ as well as NLRP3-inflammasome pathways^50^,^52^. Current and future studies are aimed at utilizing transgenic mice that are defective in specific immune pathways (specific TLR knock out mice, NLRP3 inflammasome receptor knock out mice, MyD88 receptor knock out mice), and in-vitro immune cell assays to investigate which innate immune pathway is required for the functionality of our lipid nanoparticles. Such specific understanding will enable the design and development of lipid nanoparticles as effective delivery vehicles and immune-modulators in vaccine formulations.

By modulating the charge density of the ionizable cationic lipid within the LNP, we demonstrate that the ionizable cationic lipid content is crucial for the LNP’s ability to boost immune responses to the Dengue antigen. It is possible that the reduction in LNP charge density affects the biophysical properties of DEN-80E antigen-LNP interactions and therefore might impact the LNP’s ability to boost immune responses *in-vivo*. Therefore, future studies will be aimed at evaluating the physio-chemical properties of LNP, RCL, and alternative ionizable cationic lipids when co-formulated with the DEN-80E antigens.

We did not observe prolonged, significant expression of inflammatory cytokines in mice and non-human primates injected with LNP-containing vaccine formulations. Importantly, we observed no significant increase in safety biomarkers evaluated in LNP + DEN-80E vaccinated non-human primates, including no visible reactogenicity at the local site of injection. Administration of select lipid nanoparticles and liposomes via intravenous (IV) route has been reported to lead to allergy and anaphylaxis due to complement activation^53^,^54^. Our evaluation of markers of complement activation (C3a and CBb) in the plasma of vaccinated animals at numerous time points demonstrated no significant elevation suggesting that the chosen LNP formulation, dose range of LNP, and the intramuscular route of injection (as compared to IV), supports our LNP + DEN80E formulation as a well-tolerated vaccine.

Overall, this the first study reporting a unique, tetravalent, Dengue virus sub-unit vaccine formulation containing ionizable cationic lipid nanoparticles, that is highly efficacious and well-tolerated, with the promise of serving as a next-generation Dengue virus vaccine. The level of safety and efficacy reported here warrant the need for continued evaluation of LNP containing sub-unit vaccine formulations against Dengue virus and other emerging Flaviviruses such as Zika virus.

## Materials and Methods

### Animals

Pathogen free Female BALB/c mice, and Hartley Guinea pigs were purchased from Charles River Laboratories, MA and experiments were performed in accordance with relevant guidelines from Merck’s Institutional Animal Care and Use Committee (IACUC) with pre-approved animal protocols, at the Merck Research Laboratories, West Point, PA, USA. Animals were injected intramuscularly (IM) in 50 μl (mice), or 100 μl (Guinea pigs) volume dose per quadricep (on both quadriceps) with specified antigens & adjuvants. The immunization dose regime & time line of sample collection is specified in each figure. Animal studies involving *Rhesus macaques* were performed at the New Iberia Primate Research Center (NIRC-New Iberia, LA) in accordance with relevant guidelines using protocols approved by NIRC and the Merck Research Laboratories’ Institutional Animal Care and Use Committee. Indian *Rhesus macaques* of either sex, weighing more than 3 kg, were weight and sex matched for each group. All animals were Flavivirus naïve i.e. sera negative for Dengue virus 1,2,3,4 and West Nile virus. Vaccines were administered to *Rhesus macaques* as single injection per dose, intramuscularly on the right deltoid, in a total volume of 500 μl. The composition of vaccines administered per group is listed in Fig. 6b.

### Lipid Nanoparticles, Adjuvants & Vaccine Antigens

Lipid nanoparticle (LNP), alone or encapsulating TLR9-ODN (LNP in TLR9-ODN), were prepared by rapid precipitation process as previously described^33^. The lipid components of LNP adjuvants comprised an asymmetric ionizable amino lipid (Lipid D,[47]), distearoylphosphatidylcholine (DSPC), cholesterol, and poly(ethylene glycol)2000-dimyristoylglycerol (PEG2000-DMG). LNP and LNP’s Reduced Cationic Lipid version (RCL), were prepared at varying molar ratios of the constituent lipids. LNP was prepared at a molar ratio of 58:30:10:2 ionizable amino lipid, cholesterol, DSPC and PEG-lipid^16^, while RCL was generated at a molar ratio of 33:40:25:2, respectively. For LNP encapsulating TLR9-ODN, the TLR9-ODN was incorporated at a mass ratio as defined by dose for *in-vivo* experiments. The amino lipid was synthesized at Merck (West Point, PA) according to published methods. DSPC and cholesterol were obtained from Sigma–Aldrich (St. Louis, MO). PEG2000-DMG was manufactured by NOF Corporation (White Plains, NY). Lipid nanoparticle size was determined by dynamic light scattering using a DynaPro particle sizer (Wyatt Technology, SantaBarbara, CA). Amorphous aluminum hydroxylphosphate sulfate based adjuvant termed Merck Aluminum Adjuvant (MAA), was manufactured in-house at Merck & Co., Inc. TLR9 activating agonist, referred to as TLR9-ODN throughout the paper, was originally developed by Idera pharmaceuticals and supplied in bulk under at Merck & Co., Inc^55^. Saponin based ISCOMATRIX^TM^ adjuvant was manufactured and supplied as sterilized bulk by CSL Limited, Australia under a license agreement. DEN-80E antigen for all four serotypes was expressed using stably transformed Drosophila S2 cells, and purified as previously descried^13^. For mice studies, 1 μg of DEN2-80E antigen was utilized. Tetravalent vaccine formulations of DEN1, DEN2, DEN3, DEN4 serotype specific “80E” env protein (DEN1-4-80E) content were: 3 μg, 3 μg, 3 μg, 6 μg for Guinea pig studies and 10 μg, 10 μg, 10 μg, 20 μg for *Rhesus macaques* studies.

### Safety Parameters

*Rhesus macaques* were injected with specified vaccine formulations on the right deltoid, and monitored for signs of edema and erythema by a certified veterinarian present on site at the New Iberia Primate Research Facility (NIRC), Louisiana. Local site reactogenicity was scored on a scale of 0–4 as indicated in the Draize scoring criteria in Supplementary Figure 1. Circulating safety biomarkers were evaluated from animals in all groups by collecting whole blood into serum or plasma collection tubes at indicated time points. Sera samples were utilized for assessing ALT, AST, Tbil, Dbil and C-Reactive Protein levels at indicated time points, and plasma samples were utilized for evaluation of complement activation markers, C3a and CBb. Both serum specific and plasma specific biomarkers were evaluated by established protocols in-house, at Merck Research Laboratories in West-Point, Pennsylvania, as previously described^56^,^57^,^58^.

### Cytokine & Chemokine Analyses

Sera from immunized mice and *Rhesus macaques* were collected at indicated time points. Cytokine & Chemokine responses from mouse sera were analyzed using the Quansys Biosciences (Logan, UT) 16-plex multiplex platform that consists of IL-1α, IL-1β, IL-2, IL-4, IL-6, IL-8/KC, IL-10, IL-12p70, IL-17, MCP-1, TNFα, MIP-1α, RANTES, Eotaxin/CCL11, TARC/CCL17, MDC/CCL22. As depicted in Fig. 3, pooled mouse sera from each group (n = 3–5), were assayed in triplicates. The average value of each cytokine and chemokine was calculated. The fold increases of cytokines & chemokines in treatment groups were calculated based on the PBS treated group at 4 hrs or 24 hrs time point. Cytokine & Chemokine analysis in Rhesus macaque sera was assayed using a 23-plex Luminex/EMD Millipore platform as per manufacturer’s instructions (EMD Millipore, MA). The 23-plex non-human primate panel consists of G-CSF, VEGF, TNF-α, TGF-α, sCD40L, MIP-1β, MIP-1α, MCP-1, IL-18, IL-17, IL-15, IL-13, IL-12/23 (p40), IL-10, IL-8, IL-6, IL-5, IL-4, IL-2, IL-1β, IL-1ra, IFN-γ, GM-CSF. Sera from each animal was calculated in duplicates at indicated time points. The average value of each cytokine/chemokine at 0 hr (prebleed) time point was used to calculate the fold change. The fold change at 4 hrs, 8 hrs, and 24 hrs of individual animals are depicted in Fig. 6c.

### B-cell responses

For assessing DEN2-80 specific immune responses, sera were collected from immunized mice 2 weeks post dose 3 (as depicted in Fig. 1a). Endpoint ELISA’s were utilized to assay DEN2-80E specific total IgG, IgG1 and IgG2a titres in mouse sera. Briefly, 96 well plates (NUNC #430341) were coated with overnight with 1 μg/mL of DEN2-80E antigen, blocked with a buffer containing PBS + 1% BSA (Sigma #A7030), and stained with limiting dilutions of sera from immunized mice. After washing, Goat anti-mouse IgG (Invitrogen, Carlsbad, CA, #626520), Goat anti-mouse IgG1 (S. Biotech #1070-05), or Goat anti-mouse IgG2a Abs (S. Biotech #1080-05), conjugated with HRP, were used as secondary antibodies at 1:6000 dilution. The reaction was developed by addition of TMB substrate (Virolabs, VA), and plates were read using PerkinElmer Envision plate reader measured absorbance at 450 nm.

### T cell responses

Peripheral Blood Mononuclear Cells (PBMCs) were collected from immunized *Rhesus macaques* at indicated time points, and kept frozen until use. Spleens from vaccinated mice, two weeks post dose 3, were collected and processed to isolate splenocytes. PBMCs and splenocytes were stimulated in RPMI based R10 media at 37′c, as previously described elsewhere^25^.

#### ELISPOT Assay

We performed mouse and rhesus ELIPOT assays as previously described^59^, Briefly, to each well, 4 × 10^5^ splenocytes or PBMCs were added with 2 μg/ml of DEN2-80E (mice studies) or 2 μg/ml of DEN1-80E, DEN2-80E, DEN3-80E, DEN4-80E (*Rhesus macaques studies*) specific peptide pools as indicated (15 mer overlapping by 11, custom order JPT, Germany). Spots were enumerated with an imager (AID, Germany), and the data were normalized to an input of 10^6^ cells.

#### FACS ICS Assay

1 million/well of splenocytes were cultured with 2 μg/ml of DEN2-80E peptides (15mer overlapping by 11, custom order, JPT, Germany) or equal quantity of DMSO. Mice splenocytes were cultured in the presence of Brefeldin A and anti-mouse CD28 & CD49d antibodies (BD Biosciences, CA). 6 hrs post incubation at 37′c, splenocytes were surface stained for anti-mouse-CD3(BD #557596), CD4(BD #550954), CD8BD (#563068) followed by permealization & intracellular staining for anti-mouse IFNγ (BD #557596) and TNFα (BD #557644). Rhesus macaque PBMCs were incubated with 2 μg/ml DEN1-80E, DEN2-80E, DEN3-80E, DEN4-80E specific peptide pools (15 mer overlapping by 11, custom order JPT, Germany) or equal quantity of DMSO for non stimulated controls, in the presence of Brefeldin A and anti-human CD28 & CD49d antibodies (BD Biosciences, CA). 6 hrs post incubation at 37′c, PBMCs were surface stained for anti-mouse-CD3(BD #552852), CD4(BD #562402), CD8(BD #561421) followed by permealization & intracellular staining for anti-mouse IFNγ (BD #552882), IL-2 (BD #559334) and TNFα (BD #557647). Fixed samples were then run on FACS LSRII flow cytometer (BD Biosciences) and data was analyzed using FloJo software (Treestar Inc). For calculating antigen specific multi-functional T-cell responses (Fig. 8c), we utilized the Simplified Presentation of Incredibly Complex Evaluations’ program version 5.35 (SPICE)^60^.

### Dengue Virus Neutralization Assay

Medium 199 was supplemented with 10% heat inactivated fetal bovine serum HyClone, Logan, UT), glutamine (Mediatech, New Jersey) and penicillin-streptomycin (Mediatech, New Jersey) for vero cell maintenance (ATCC-CCL-81, Virginia). The Dengue virus strains used in neutralization assays were: DENV-1 (strain WestPac-74), DENV-2 (strain S18603), DENV-3 (strainCH53489), and DENV-4 (strain TVP-360). Dengue viruses were kindly provided by Alan Barrett (University of Texas Medical Branch, Galveston, TX). Vero cells were utilized to expand viruses at low multiplicity of infection, and supernatants from infected cells were collected, clarified, and frozen until use for neutralization assays, as described previously^14^.

Dengue virus neutralization titres were assayed using an infra-red dye (IRD) based high-throughput and sensitive neutralization assay that was developed and well-established by by our group (described in detail^18^,^61^). Briefly, vero cells were seeded at 1.5 × 10^4 cells/well in a 96wp format. 1000 pfu/ml of DEN1–4 virus was pre-incubated with dilutions of heat-inactivated sera from immunized mice, Guinea pigs, and Rhesus macaques, for 1 hr at 37′C, and added on to vero cells. 4 days later cells were fixed, and incubated with anti-Dengue specific 4G2 mouse antibody (SDIX) followed by biotinylated anti-mouse IgG antibody Catalogue no. BA-2000) (Vector labs Inc., Burlingame, CA), followed by a cocktail of DRAQ5 dye (Cell Signaling, MA), and IRDye 800CW Streptavidin (Li-Cor). Stained plates were washed, air-dried and were read using an infrared Odyssey SA imaging system (Li-Cor Biosciences, NE). Serum end-point neutralization titers or NT50 values calculated i.e. the reciprocal of the highest serum dilution that reduces the 800 nm/700 nm fluorescence integrated intensity ratio greater than 50% when compared to virus control included on each assay plate. If a sample failed to neutralize at this dilution, a titer of 1:5 was assigned to that sample and this dilution was used to calculate the geometric mean of that group.

### Statistical analysis

Statistical analyses were performed by either the One-Way ANOVA test with Bonneferri’s multiple comparison post hoc tests or by unpaired student T-test test using GraphPad 4.03 (GraphPad, San Diego, CA, USA). Data were expressed as mean ± standard error of the mean (SEM) or median ± quartiles. Differences between treatment groups and controls were considered statistically significant at p < 0.001 or p < 0.05. The number of mice, Guinea pigs, and Non-human primates used per assay is indicated in the figure legends.

## Additional Information

**How to cite this article**: Swaminathan, G. *et al*. A Tetravalent Sub-unit Dengue Vaccine Formulated with Ionizable Cationic Lipid Nanoparticle induces Significant Immune Responses in Rodents and Non-Human Primates. *Sci. Rep.* **6**, 34215; doi: 10.1038/srep34215 (2016).

## Supplementary Material

Supplementary Information
