# Supplementary material for: A Tetravalent Sub-unit Dengue Vaccine Formulated with Ionizable Cationic Lipid Nanoparticle induces Significant Immune Responses in Rodents and Non-Human Primates
Source: Sci Rep. 2016 Oct 5;6:34215. doi: 10.1038/srep34215 (PMC5050434; doi:10.1038/srep34215)
Supplement: Supplementary Information [file srep34215-s1.doc]

**Supplementary Information**

**A Tetravalent Sub-unit Dengue Vaccine Formulated with Ionizable Cationic Lipid Nanoparticle induces Significant Immune Responses in Rodents and Non-Human Primates**

Gokul Swaminathan^1^, Elizabeth A. Thoryk^1^, Kara S. Cox^1^, Jeffrey S. Smith^2^, Jayanthi J. Wolf^3^, Marian E. Gindy^2^, Danilo R. Casimiro^1^ and Andrew J. Bett*^1^

**Supplementary Figure and Figure Legends:**

**Supp Fig 1:**

**
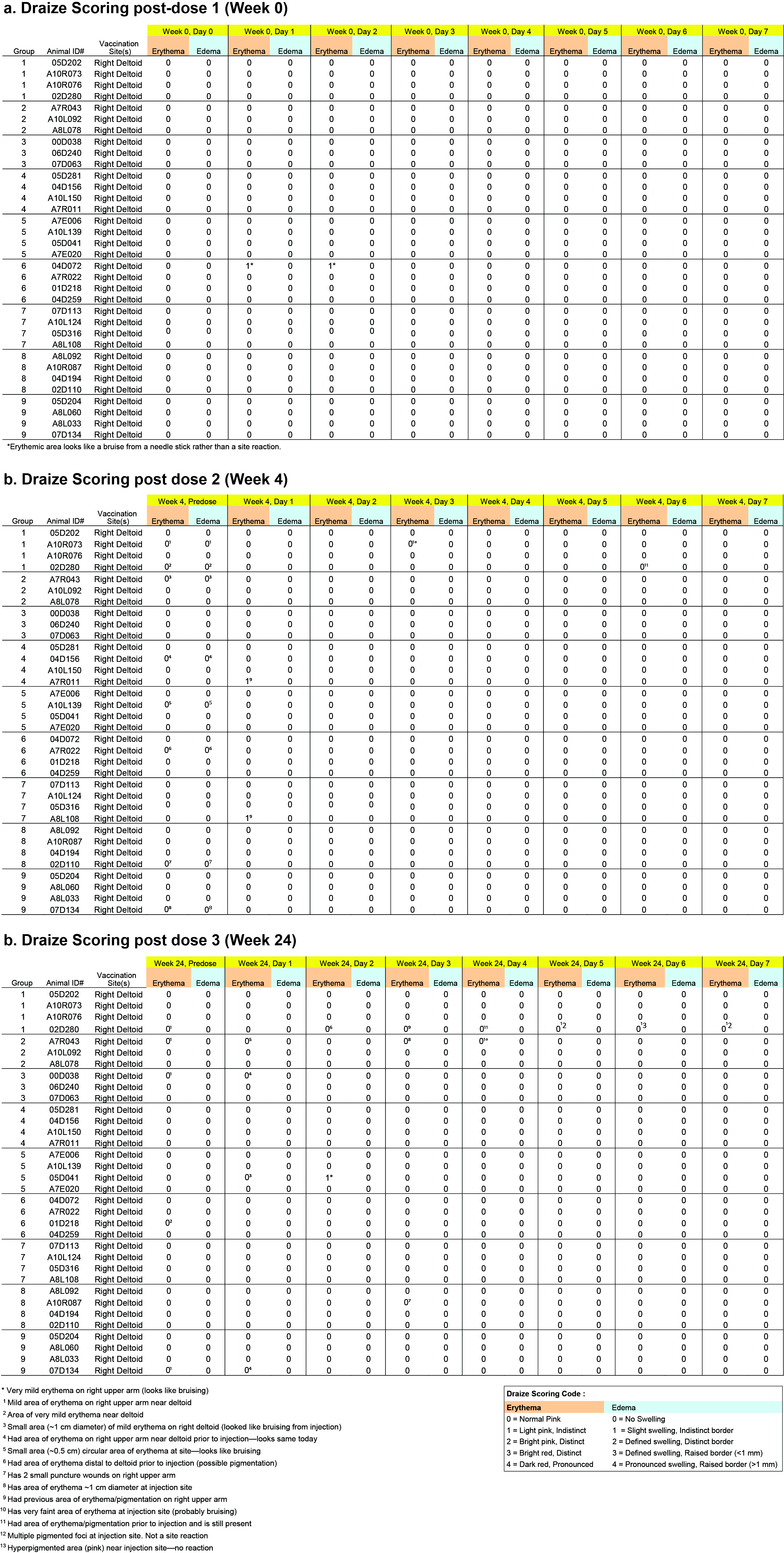
**

**Suppl Figure 1.** Draize Scoring in Immunized Rhesus macaques:

Local site reactogenicity including signs of erythema and edema were evaluated in Rhesus macaques in all study groups, everyday, for seven days, post-dose 1 (S1A), post-dose 2 (S1B), and post-dose 3 (S1C). The outcome was tabulated on a scale of 0 to 4, as defined by an established Draize score criteri

**Supp Fig 2:**


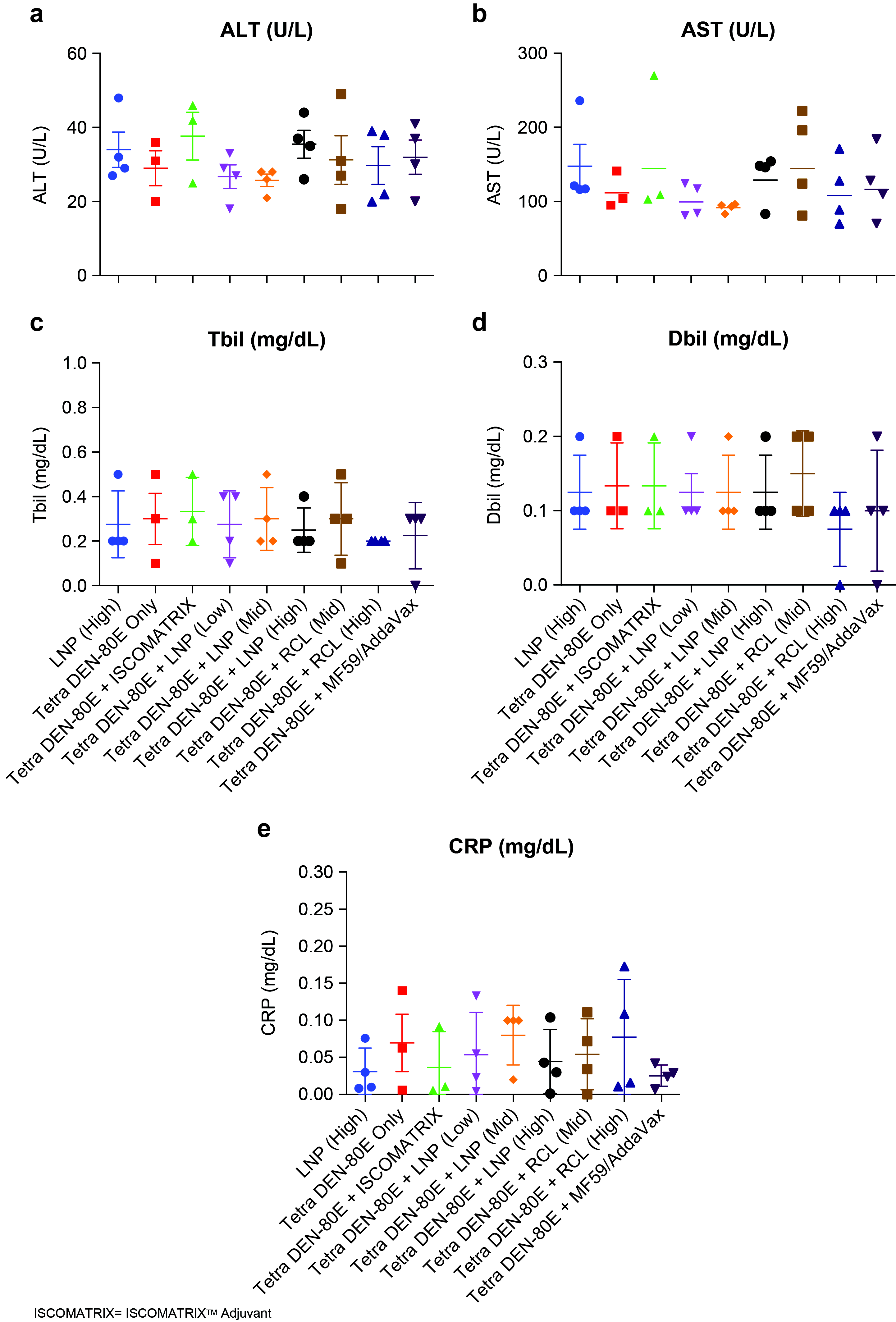


**Suppl Figure 2.** Safety Biomarkers in Immunized Rhesus macaques:

Circulating safety biomarkers were evaluated from animals in all study groups by collecting serum at 24hrs post dose 1. Sera samples were utilized for assessing ALT (S2A), AST (S2B), Tbil (S2C), Dbil (S2D), and C-Reactive Protein (CRP) (S2E) levels, measured in indicated measuring units. Each dot/symbol represents values from individual animal per group.

**Supp Fig 3:**


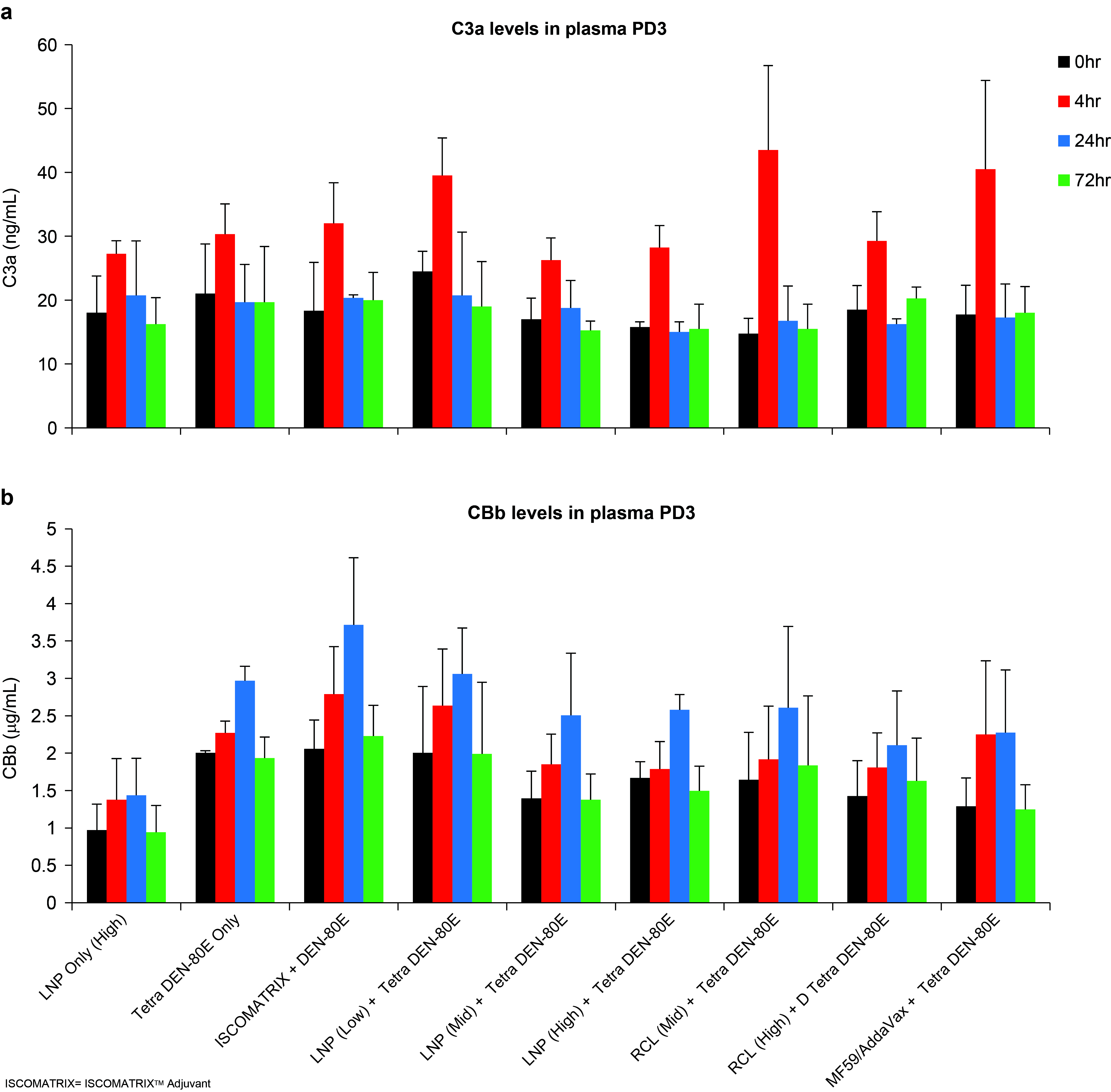


**Suppl Figure 3.** Complement activation in Immunized Rhesus macaques:

Whole blood from all animals in the study group was collected in Plasma/EDTA collection tubes at 0hrs (prebleed) or 4hrs, 24hrs and 72hrs post-dose 3. Plasma samples were utilized for evaluation of complement activation markers, C3a (S3A) and CBb (S3B) at indicated time points in designated measuring units. Each bar represents average values from 3-4 animals/group assayed in duplicates.

**Supp Fig 4:**


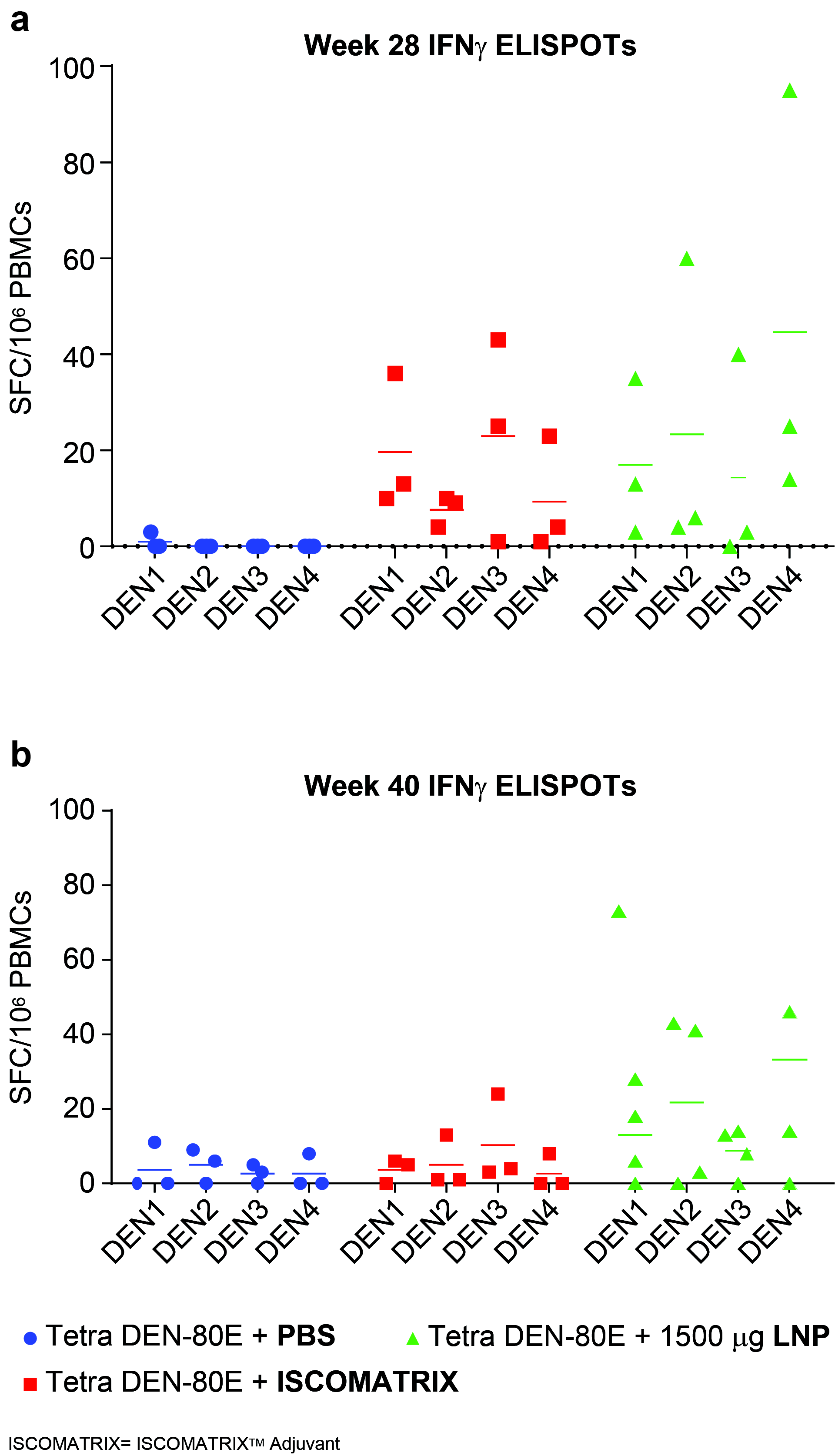


**Suppl Figure 4**.T cell responses in Immunized Rhesus macaques:

At week 28 (S4A), and at week 40 (S4B), Peripheral Blood Mononuclear Cells (PBMCs) were collected from immunized animals (from specified groups) and incubated with DEN1-80E, DEN2-80E, DEN3-80E, DEN4-80E specific peptide pools, and subjected to IFN-γ ELISPOT assay. IFN-γ producing spots per million (SFU) PBMCs were determined against each DEN serotype specific peptides. Each dot/symbol represents values from individual animal per group.
